# Exploring the Views and Experiences of Moral Distress Among Newly Graduated Registered Nurses in Saudi Arabia: A Sequential Explanatory Mixed-Methods Study Protocol

**DOI:** 10.3390/nursrep16070240

**Published:** 2026-07-10

**Authors:** Hanan Alfaifi, Michael Brown, Clare McVeigh, Lynne Marsh

**Affiliations:** 1 School of Nursing and Midwifery, Queen’s University Belfast, Belfast BT9 7BL, UK; m.j.brown@qub.ac.uk (M.B.); clare.mcveigh@qub.ac.uk (C.M.); l.marsh@qub.ac.uk (L.M.); 2 Nursing College, Najran University, Najran 55461, Saudi Arabia

**Keywords:** moral distress, coping, tertiary care, newly graduated registered nurses, nursing

## Abstract

**Background**: Moral distress is a significant concern in nursing, particularly among newly graduated registered nurses transitioning into professional practice. Limited experience, high workloads, and exposure to ethically challenging situations may negatively affect well-being and contribute to early-career attrition. Evidence on how NGRNs experience and manage moral distress globally and in Saudi Arabia remains limited, and the role of healthcare organisational factors in shaping this phenomenon is not yet well understood. **Aim**: This study aims to examine the experiences of NGRNs regarding moral distress in adult tertiary care hospitals in Saudi Arabia, focusing on contributing factors, coping strategies, and organisational influences. **Methods**: A mixed-methods sequential explanatory design will be used. In the quantitative phase, NGRNs will complete the Measure of Moral Distress—Healthcare Professionals (MMD-HP) and a demographic questionnaire. Data will be analysed using descriptive and inferential statistics to assess levels of moral distress and associations with intention to leave. The qualitative phase will involve semi-structured interviews with NGRNs and focus groups with nursing managers, which will be analysed using reflexive thematic analysis. Integration will occur at the data collection and interpretation stages. The study is guided by Bronfenbrenner’s Ecological Systems Theory. Ethical approval has been obtained from Queen’s University Belfast and the Najran Health Cluster. **Expected Impact**: The study is expected to provide a comprehensive understanding of moral distress among NGRNs and inform the development of targeted organisational strategies to support Registered Nurse wellbeing, retention, and quality of care.

## 1. Introduction and Significance of the Study

Registered nurses constitute the largest proportion of the global healthcare workforce, accounting for approximately 59% of all health professionals worldwide [1]. In recent years, the nursing profession has expanded considerably and remains a cornerstone of healthcare delivery across diverse clinical settings [2]. The role of RNs requires a high level of commitment, as they provide continuous patient care while managing role conflict and fulfilling multiple responsibilities, including caregiving, education, and patient advocacy [3]. These demands contribute to the increasingly complex nature of nursing practice.

A major challenge facing healthcare systems globally is the shortage of RNs, which was estimated at approximately 5.9 million prior to the COVID-19 pandemic [4]. To meet future healthcare needs, the International Council of Nurses projects that more than 13 million additional nurses will be required worldwide by 2030 [4]. This challenge is particularly evident in the Gulf Cooperation Council (GCC) countries—namely Saudi Arabia, the United Arab Emirates, Oman, Bahrain, Qatar, and Kuwait—where healthcare systems have historically relied heavily on expatriate nurses. In these countries, local citizens constitute only 20–35% of the nursing workforce [4].

In Saudi Arabia, high turnover rates and intentions to leave the profession continue to place pressure on the healthcare system [5]. Evidence suggests that nurses’ decisions to remain in or leave their roles are closely associated with their quality of life, including both psychological and physical well-being [6]. At the same time, increasing workloads, staffing shortages, and growing clinical demands require nurses to possess a broad range of competencies to deliver safe and effective care [3].

Contemporary nursing practice is increasingly characterised by growing organisational demands and complex care environments that require nurses to balance direct patient care with competing clinical and organisational responsibilities. These demands contribute to the complexity of nursing practice and require nurses to balance competing priorities while delivering safe, high-quality care [7]. Such complex care environments may increase nurses’ exposure to ethically challenging situations, thereby contributing to moral distress [8]. Consequently, moral distress has become an increasingly important concern within nursing practice. It is widely recognised as a common experience across clinical settings and has been reported among healthcare professionals working in a range of specialities and care environments [9].

The concept of moral distress was first introduced by Jameton [10], who defined it as the experience of knowing the ethically appropriate action but being unable to act upon it because of institutional or organisational constraints. In nursing practice, adherence to professional and ethical standards is fundamental, and situations that conflict with personal and professional values may give rise to moral distress [11]. Such situations may occur in environments characterised by inadequate care delivery, prolonged patient suffering, insufficiently skilled staff, and limited organisational support, including issues related to workload, working conditions, and compensation [11].

Moral distress is often associated with emotional responses such as frustration, grief, and powerlessness, particularly among nurses working in tertiary care settings [12]. Over time, these experiences may contribute to workplace disengagement, reduced confidence, loss of professional purpose, and burnout [13]. Coping mechanisms, defined as the conscious use of cognitive and behavioural strategies to manage stressors, play an important role in mitigating the effects of moral distress [14]. These strategies may be strengthened through ethical education and accumulated clinical experience [15].

NGRNs represent a particularly vulnerable group within the nursing workforce and often experience high turnover rates during the early years of practice [16]. Newly graduated RNs are commonly defined as nurses within their first three years after graduation [17]. Within Benner’s novice-to-expert framework, they may be considered novice or advanced beginner practitioners as they begin to develop clinical experience and professional competence [18]. This developmental stage is characterised by limited clinical experience and the gradual development of clinical judgement and confidence.

The transition from student to practising nurse is frequently accompanied by stress, fatigue, professional insecurity, and challenges in adapting to workplace demands [19,20]. Limited clinical experience may reduce NGRNs’ confidence in responding to ethically complex situations, increasing their vulnerability to moral distress and its consequences [16,20]. In Saudi Arabia, NGRNs have reported fear and uncertainty during the transition to practice, often resulting from a mismatch between expectations and the realities of professional nursing roles [21].

Moral distress has been identified as an important factor affecting both the well-being and retention of NGRNs [22]. For example, approximately 17.5% of NGRNs in the United States leave their positions within the first year, with moral distress identified as a contributing factor. Similarly, moral distress has been associated with healthcare professionals’ intentions to leave their jobs in Saudi Arabia [23]. These findings highlight the importance of organisational support during the transition to practice, including mentorship, clinical supervision, constructive feedback, and opportunities for professional development [24].

Although moral distress has been widely studied within the nursing profession, existing research has largely focused on experienced nurses or specific clinical specialities. Several studies have examined the causes of moral distress and strategies to address it [7,11,25]; however, limited evidence specifically examines how NGRNs experience and navigate moral distress in clinical practice. Furthermore, little is known about the coping strategies used by NGRNs or the organisational approaches that may help address moral distress within this population.

Therefore, this study aims to explore the views and experiences of newly graduated registered nurses regarding moral distress in adult tertiary care hospitals in Saudi Arabia, with a particular focus on contributing factors, coping strategies, and the organisational role in addressing moral distress.

### 1.1. Study Design

This study will adopt a sequential explanatory mixed-methods design comprising two phases, beginning with a quantitative component followed by a qualitative phase. The integration of quantitative and qualitative approaches is expected to generate a more comprehensive understanding of the research phenomenon by combining numerical measurement with in-depth exploration [26].

The rationale for employing this design is to broaden the scope of inquiry and deepen interpretation by allowing findings from one phase to inform the next [27]. The quantitative phase is intended to provide an overview of moral distress among NGRNs, identify situations commonly associated with moral distress, and explore differences across participant characteristics. Given that moral distress is not a widely recognised concept among many nurses, the Measure of Moral Distress—Healthcare Professionals (MMD-HP) will provide contextual information regarding morally distressing situations and encourage reflection on experiences that may subsequently be explored in greater depth during interviews.

Quantitative findings will guide participant selection and refine interview questions, thereby supporting a more focused exploration of moral distress. Accordingly, the quantitative phase is intended to provide contextual understanding and inform qualitative inquiry rather than develop predictive models of moral distress.

**Figure 1:**
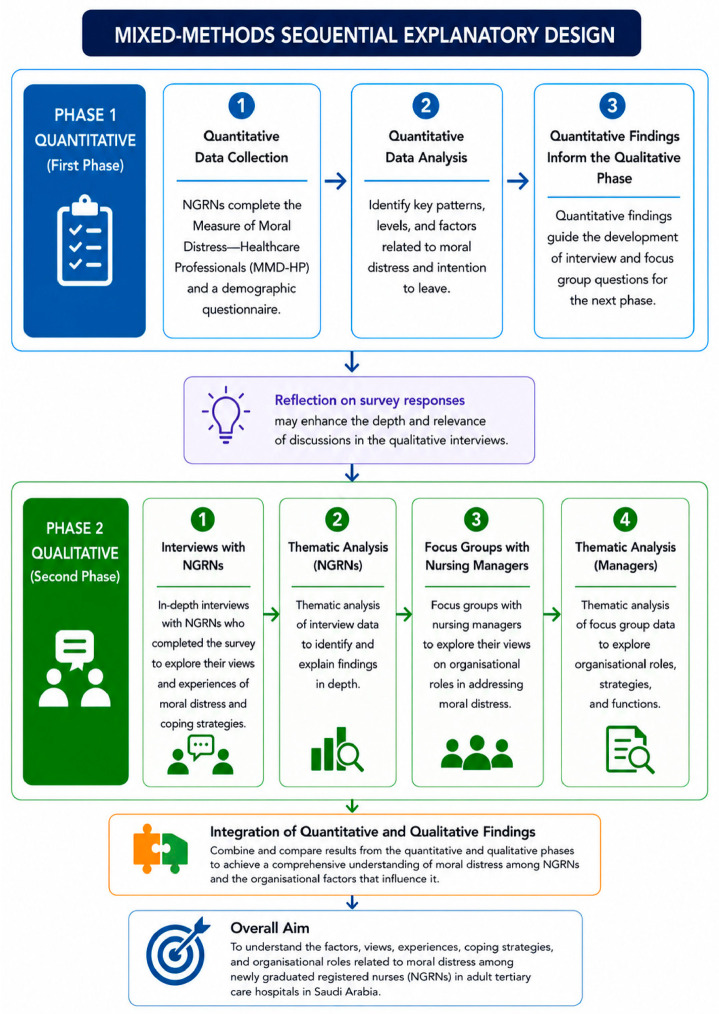
Summary of the study phases, key processes, and participants.

The primary aim of this study is not only to identify factors contributing to moral distress but also to gain a deeper understanding of the views, experiences, and coping strategies of NGRNs regarding moral distress in adult tertiary care settings. Figure 1 provides an overview of the study phases.

#### Aim and Research Questions

To explore the views, experiences, and coping strategies of NGRNs regarding moral distress in adult tertiary care hospitals in Saudi Arabia and to examine the organisational role in addressing moral distress.

### 1.2. Theoretical Framework

**Figure 2:**
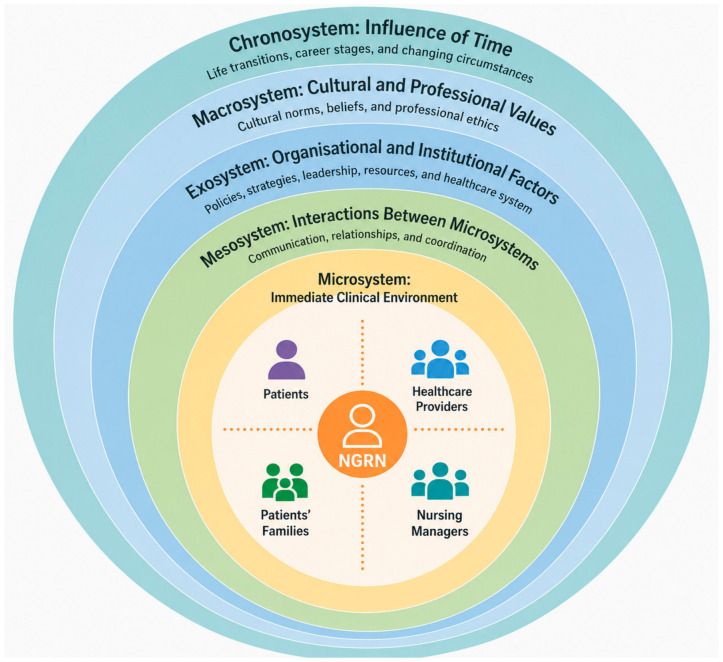
Adapted Bronfenbrenner Ecological Systems Model for NGRNs.

This study is guided by Urie Bronfenbrenner’s Ecological Systems Theory [28], which conceptualises human experiences as the result of dynamic interactions between individuals and multiple layers of their environment. The theory proposes that behaviour and experiences are shaped by interconnected systems that range from immediate settings to broader societal influences (Figure 2). Bronfenbrenner’s Ecological Systems Theory will provide a conceptual lens for understanding the multiple influences that may contribute to moral distress among NGRNs. The framework will support the interpretation of findings by considering factors operating at individual, interpersonal, organisational, and broader contextual levels. While the framework will inform the overall understanding of moral distress, data collection and analysis will remain guided by the study’s research questions and the principles of reflexive thematic analysis.

In this study, moral distress is conceptualised as a multi-level phenomenon arising from nurses’ interactions within these systems. The microsystem refers to the immediate clinical environment in which NGRNs operate, including direct interactions with patients, colleagues, and supervisors. The mesosystem encompasses the interactions among these immediate contexts, such as communication and collaboration among healthcare team members, that may influence ethical decision-making and contribute to moral distress.

The exosystem encompasses organisational structures and institutional factors that indirectly affect nurses’ experiences, including staffing levels, hospital policies, management practices, and availability of support. The macrosystem reflects broader cultural, ethical, and professional values that shape expectations of nursing practice, particularly within the Saudi Arabian context. Finally, the chronosystem captures the influence of time, including the transition from student to professional nurse, which may intensify vulnerability to moral distress during early career stages. Applying this framework will enable a comprehensive understanding of moral distress across individual, interpersonal, organisational, and societal levels, supporting interpretation of the findings within a broader contextual perspective.

### 1.3. Setting

The study will be conducted in two major tertiary hospitals affiliated with the Ministry of Health in Najran, Saudi Arabia. Data will be collected across multiple adult tertiary care departments, including intensive care units, coronary care units, emergency departments, oncology units, medical wards, surgical wards, and artificial kidney units. Adult tertiary care settings have been selected due to the complexity of care and evidence of early placement of NGRNs in these settings following completion of their internships.

### 1.4. Participants and Sampling

The study population will comprise NGRNs and nursing managers. Newly graduated RNs were defined as nurses with up to three years of clinical experience who were actively engaged in clinical practice [17]. Nursing managers will include preceptors, head nurses, nursing educators, and nursing directors who supported and supervised NGRNs.

Different sampling strategies will be applied across the study phases. In the quantitative phase, total population sampling will be used, with all eligible NGRNs across the participating hospitals invited to participate. The estimated accessible population is approximately 90 NGRNs. The researcher will aim to recruit at least 60% of the accessible population (approximately 54 respondents). This recruitment target is considered sufficient for the planned descriptive and exploratory inferential analyses [29], including estimating levels of moral distress, examining patterns across MMD-HP items, and exploring associations with intention to leave.

For the qualitative phase, an initial sample of approximately 15 NGRNs will be sought for individual interviews. Participants will be purposively selected from survey respondents using a maximum variation sampling strategy to ensure diversity in demographic and professional characteristics and experiences of moral distress. Nursing managers will participate in focus groups comprising 3–5 participants, with additional groups conducted as required. Recruitment and data collection will continue iteratively until data saturation is achieved, defined as the point at which additional interviews no longer yield meaningful insights relevant to the research aims, as determined through ongoing preliminary data analysis.

## 2. Pilot Testing

Prior to data collection, the survey procedures, demographic questionnaire, and interview guide will be pilot tested with a small number of nurses who meet the study inclusion criteria. Pilot testing will be undertaken to assess clarity, relevance, and feasibility, and amendments will be made where necessary. Data collected during pilot testing will not be included in the main study.

### 2.1. Recruitment

Recruitment will be facilitated by nursing coordinators, who will serve as gatekeepers at the participating hospitals. Study materials, including invitation letters, participant information sheets, consent forms, and survey links, will be distributed electronically to eligible participants. Participation will be entirely voluntary.

Participants will access the quantitative survey via QR codes displayed on recruitment posters and in electronic communications. Completion of the survey will be considered implied consent to participate in the quantitative phase.

At the end of the survey, participants will be invited to indicate their willingness to participate in the qualitative phase. Those who express interest will be asked to provide contact details separately to maintain confidentiality. The researcher will then contact these participants directly to arrange individual interviews or focus group participation at a mutually convenient time.

### 2.2. Inclusion and Exclusion Criteria

Newly graduated RNs will be eligible for inclusion if they are registered nurses with up to three years of clinical experience, are currently working in clinical settings, and are able to communicate in either Arabic or English. Nurses with more than three years of experience, those not engaged in direct clinical practice, and nursing students or interns will be excluded.

**Figure 3:**
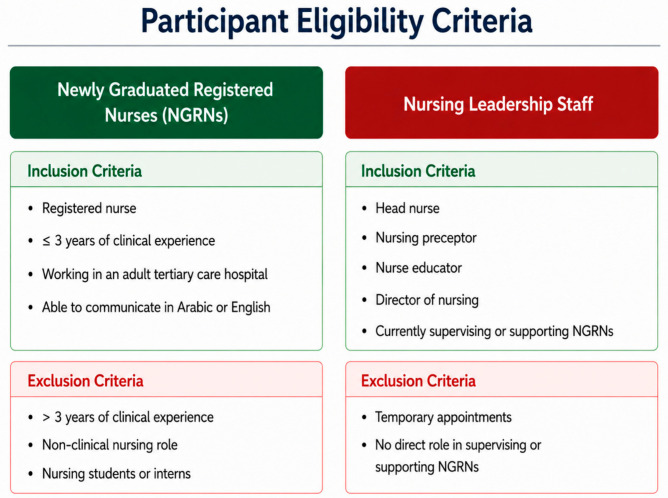
Summary of inclusion and exclusion criteria for NGRNs and nursing managers.

Nursing managers will be eligible if they hold roles such as preceptors, head nurses, nurse educators, or nursing directors and are actively involved in supporting or supervising NGRNs. Individuals in temporary, administrative-only, or non-relevant leadership roles will be excluded. See the summary below (Figure 3).

### 2.3. Data Collection

#### 2.3.1. Quantitative Phase

Quantitative data will be collected using the Measure of Moral Distress—Healthcare Professionals (MMD-HP), a validated instrument comprising 27 items designed to assess both the frequency and intensity of moral distress. Permission to use the scale has been sought from the authors [30]. Participants will rate each item using Likert-type scales, and composite scores will be calculated by multiplying frequency and intensity scores. Total scores will range from 0 to 432, with higher scores indicating greater levels of moral distress. The original English version of the MMD-HP will be used in this study. Translation will not be undertaken because nursing education, documentation, and professional communication in the study setting will be conducted in English. Consequently, the original instrument was considered appropriate for the target population. The scale will be administered electronically via a secure online platform and is expected to take approximately 10–15 min to complete. In addition to the MMD-HP, demographic information will be collected, including age, gender, nationality, years of experience, educational level, shift patterns, and clinical unit. The scale also includes items assessing participants’ considerations of leaving their position due to moral distress, which will be analysed as part of the study outcomes.

#### 2.3.2. Qualitative Phase

Qualitative data will be collected through semi-structured individual interviews with NGRNs and focus groups with nursing managers. An interview guide will be developed based on the study objectives, the relevant literature, and findings from the quantitative phase. The interviews and focus groups will explore participants’ views, experiences, coping strategies, and organisational perspectives related to moral distress.

Interviews and focus groups will be conducted either face-to-face or via secure online platforms, depending on participant preference and availability. Each interview is expected to last approximately 30–60 min, while focus groups will last between 45–90 min. All sessions will be audio-recorded with participants’ consent.

**Figure 4:**
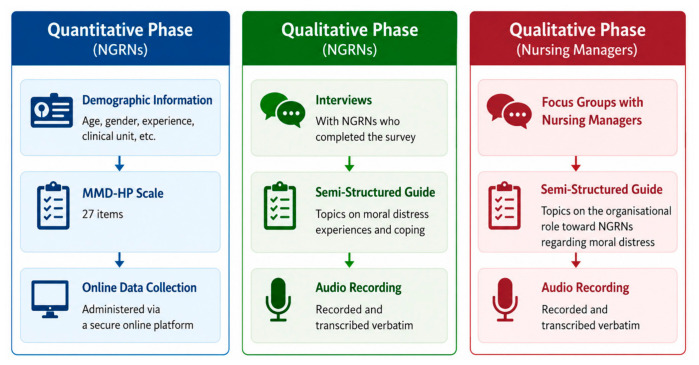
Data collection process.

Audio recordings will be transcribed verbatim. Interviews conducted in Arabic will be translated into English, and the translations will be verified by an independent bilingual expert to ensure accuracy and consistency. All data will be anonymised prior to analysis to maintain participant confidentiality; see Figure 4, which illustrates the data collection process.

### 2.4. Data Analysis

#### 2.4.1. Quantitative Analysis

Quantitative data will be analysed using the Statistical Package for the Social Sciences (SPSS) software (version 31; IBM Corp., Armonk, NY, USA). Descriptive statistics, including frequencies, percentages, means, and standard deviations, will be used to summarise demographic characteristics, levels of moral distress, and participants’ responses regarding intention to leave their position due to moral distress.

The distribution of continuous variables will be examined using graphical methods and measures of skewness and kurtosis prior to conducting inferential analyses. Inferential statistical analyses will be conducted to explore differences in moral distress scores across participant characteristics and intention-to-leave status. Independent-samples *t*-tests and one-way analysis of variance (ANOVA) will be used, where appropriate, depending on the nature and categorisation of the variables.

Given that the quantitative phase is intended to provide contextual understanding of moral distress and inform the subsequent qualitative phase, rather than develop predictive models, multivariable analyses are not planned. The electronic survey will require completion of all questionnaire items prior to submission; therefore, missing data are unlikely. Statistical significance will be set at *p* < 0.05. Effect sizes and 95% confidence intervals will also be reported alongside inferential statistical analyses to facilitate interpretation of the magnitude and precision of the findings.

#### 2.4.2. Qualitative Analysis

Qualitative data will be analysed using Braun and Clarke’s six-step reflexive thematic analysis approach [31]. This will involve data familiarisation, generation of initial codes, searching for themes, reviewing themes, defining and naming themes, and producing the final report. NVivo (version 15) software will be used to facilitate data organisation and coding. Data analysis will be conducted iteratively, with codes and themes refined throughout the analytic process. Consistent with a reflexive thematic analysis approach, coding will primarily be undertaken by the researcher, with regular discussions with the supervisory team to support critical reflection and theme development rather than achieve coding consensus.

Researcher reflexivity will be maintained throughout the study through ongoing reflection on assumptions, experiences, and potential influences on data interpretation. Reflexive notes will be documented during data collection and analysis to support transparency and analytical rigour.

## 3. Integration

Data integration will occur at two stages. During the data collection phase, quantitative findings will inform the development of the qualitative interview guide and participant selection (connecting), allowing key areas identified in the survey to be explored in greater depth. During the analysis phase, quantitative and qualitative findings will be integrated through comparison and interpretation (merging), whereby results from both phases will be examined together to identify areas of convergence, complementarity, and divergence [32]. Where findings diverge, these differences will be explored to identify potential contextual, organisational, or experiential factors that may explain inconsistencies between quantitative and qualitative results. Integration is intended to enable a more comprehensive understanding of moral distress by examining how qualitative findings explain, expand upon, and contextualise quantitative results. This integrated approach will provide a richer understanding of moral distress among NGRNs than either method could achieve independently.

### 3.1. Rigour

Quantitative rigour will be established through adherence to the principles of validity, reliability, objectivity, and generalisability [33]. Validity refers to the extent to which an instrument accurately measures the intended construct [34]. The Measure of Moral Distress—Healthcare Professionals (MMD-HP) has been widely validated and used across diverse healthcare settings to assess moral distress [30].

Reliability concerns the consistency of measurement, indicating the instrument’s ability to produce stable results under similar conditions [34]. The MMD-HP has demonstrated high internal consistency, with a reported Cronbach’s alpha of 0.93 [30].

Objectivity will be maintained by adhering to standardised data collection procedures and relying on statistical analysis to minimise researcher bias [35].

Generalisability refers to the extent to which findings can be applied beyond the study sample [36]. This will be supported through clearly defined inclusion and exclusion criteria, a validated measurement tool, and transparent reporting of statistical procedures and results [36].

Qualitative rigour will be guided by the criteria proposed by Lincoln and Guba, including credibility, dependability, confirmability, and transferability [37]. To enhance credibility, strategies such as in-depth exploration of participants’ experiences will be employed. This requires prolonged engagement in the field to facilitate a deeper understanding of the cultural and social context surrounding the phenomenon of interest [38]. Interviews are expected to last approximately one hour, and data collection will span about three months to ensure sufficient engagement. Transferability is addressed by presenting rich, detailed data that enable readers to assess the extent to which the findings are applicable to other contexts [39]. This will be accomplished through comprehensive descriptions of the research methods, study context, participants, and phenomenon under investigation, thereby providing a “thick description” that facilitates an in-depth understanding of the study setting. This will enable readers to determine the relevance of the findings to similar tertiary healthcare settings and newly graduated nursing populations. Dependability and confirmability will be supported by maintaining a clear audit trail that documents all stages of data collection and analysis, alongside regular reflective practices to acknowledge and minimise potential researcher bias [40].

Data triangulation will be achieved by incorporating perspectives from both newly graduated registered nurses and nursing managers, allowing for a more comprehensive understanding of the phenomenon [41]. Reflexivity will be maintained throughout the study to enhance transparency and analytical rigour. The researcher will engage in ongoing reflection regarding personal assumptions, perspectives, and potential influences on data collection and interpretation. Reflexive notes will be recorded throughout the research process and used to support critical engagement with the data and the development of themes.

### 3.2. Ethical Considerations

Ethical approval has been obtained from the Research Ethics Committee at Queen’s University Belfast and the Institutional Review Board of Najran Health Cluster. The study will be conducted in accordance with the principles of the Declaration of Helsinki. Informed consent will be obtained from all participants prior to participation. Participation will be entirely voluntary, and participants will be informed of their right to withdraw from the study at any time without consequence.

Confidentiality and anonymity will be ensured through unique identification codes and secure data storage in password-protected systems accessible only to the research team. A distress protocol will be implemented to manage any potential emotional discomfort that may arise during interviews. Participants will be provided with information about appropriate support services should they require additional support.

## 4. Results

This study is currently ongoing at the time of submission, and therefore, no findings are reported in this manuscript.

## 5. Discussion

Moral distress is increasingly recognised as a significant challenge in nursing practice, with implications for nurses’ well-being, job satisfaction, retention, and the quality of patient care. Newly graduated RNs may be particularly vulnerable during the transition from student to professional nurse, as they encounter ethically challenging situations while continuing to develop clinical competence and professional confidence. Despite growing international interest in moral distress, limited research has examined how NGRNs experience and navigate it in clinical practice, particularly in the Saudi Arabian context.

This study seeks to address this gap through a sequential explanatory mixed-methods design that examines the extent, contributing factors, coping strategies, and organisational influences associated with moral distress among NGRNs working in adult tertiary care hospitals. By integrating quantitative measurement with in-depth qualitative exploration, the study is designed to provide both an overview of moral distress and a deeper understanding of how it is experienced, interpreted, and managed in practice.

A key strength of the study is the integration of quantitative and qualitative approaches, enabling survey findings to inform qualitative inquiry and contribute to a more comprehensive understanding of moral distress. The inclusion of nursing managers further strengthens the study by incorporating organisational perspectives on factors that may influence NGRNs’ experiences. In addition, Bronfenbrenner’s Ecological Systems Theory provides a framework for interpreting findings across multiple levels of influence, including individual, interpersonal, organisational, and broader socio-cultural factors.

The study has the potential to contribute to nursing knowledge by advancing understanding of the factors associated with moral distress among NGRNs and the strategies used to manage it. The findings may help to inform the development of organisational initiatives, such as supportive leadership practices, ethical education, mentorship programmes, and workplace policies aimed at supporting NGRNs during their transition to professional practice.

Several methodological considerations should be acknowledged. Although all eligible NGRNs across the participating hospitals will be invited to participate in the quantitative phase, participation will remain voluntary, and individuals with particularly strong experiences or views regarding moral distress may be more likely to participate, potentially introducing selection bias. Furthermore, the study will be conducted in two tertiary hospitals within a single health cluster in Saudi Arabia, which may limit the transferability of the findings to other healthcare settings and regions within the country. However, detailed descriptions of the study context and participants will be provided to help readers assess the applicability of the findings to their own settings.

Overall, this protocol outlines a rigorous and theoretically informed approach to investigating moral distress among NGRNs. The study has the potential to generate evidence that may inform nursing practice, organisational policy, and future research aimed at supporting the well-being and professional sustainability of NGRNs.

## 6. Conclusions

This study seeks to contribute to a deeper understanding of moral distress among NGRNs by examining the causes, factors, experiences, coping strategies, and organisational influences. Using a mixed-methods approach, the study is designed to provide both measurable insights and in-depth perspectives on this complex phenomenon in adult tertiary care settings. The study has the potential to inform the development of organisational policies and support systems to enhance the well-being of NGRNs, facilitate early-career transitions, and improve the quality and safety of patient care.

## Data Availability

The data generated during this study will be available on request from the corresponding author due to privacy and ethical restrictions.

## Public Involvement Statement

There was no public involvement in any aspect of this research.

## Guidelines and Standards Statement

This manuscript was prepared in accordance with the Good Reporting of a Mixed Methods Study (GRAMMS) guidelines for mixed-methods research.
